# Synergistic Anti‐Obesity Effect of *Akkermansia muciniphila* AKM Lab‐01 and *Garcinia cambogia* Extract via Gut Microbiota Remodeling in Diet‐Induced Obese Mice

**DOI:** 10.1002/fsn3.72140

**Published:** 2026-07-28

**Authors:** Baojia Huang, Zhipeng Chen, Wenbin Xue, Zilun Pu, Yalin Zhou, Sherlyn Sze Ning Koay, Ping Kong, Yingying Zhao, Lihong Tai, Zhou Lan, Yibo Xian, Amanda Juan Chen

**Affiliations:** ^1^ Moon (Guangzhou) Biotech Co. Ltd. Guangzhou Guangdong China; ^2^ Department of Chemical Engineering Tsinghua University Beijing China; ^3^ Key Lab of Industrial Biocatalysis, Ministry of Education Tsinghua University Beijing China

**Keywords:** AKM Lab‐01, Anti‐obesity, *Garcinia cambogia* extract, Gut microbiota, Pasteurized *Akkermansia muciniphila*

## Abstract

Obesity is a global health crisis driven by complex metabolic dysregulation. Although *Akkermansia muciniphila* (AKK) has emerged as a promising next‐generation probiotic for metabolic health, its synergistic potential with natural anti‐obesity compounds remains largely unexplored. Here, we evaluated the combined administration of pasteurized *A. muciniphila* (AKM Lab‐01) and *Garcinia cambogia* extract (GCE) in a mouse model of high‐fat diet‐induced obesity. The combination treatment significantly ameliorated obesity‐related phenotypes, including reduced body weight, decreased fat mass, improved serum metabolic parameters, and attenuated adipose tissue inflammation. Adipose tissue transcriptomic profiling revealed enhanced lipid catabolism and downregulation of pro‐inflammatory pathways. Metagenomic sequencing showed marked gut microbiota remodeling, characterized by increased abundance of *Lactococcus* and decreased levels of *Clostridium* and *Eisenbergiella*. Integrated correlation analysis linked these microbial shifts to transcriptional reprogramming in adipose tissue. Using a 3 T3‐L1 adipocyte model, we further confirmed that *Lactococcus* plays a potential role in regulating lipid metabolism and inflammation. Collectively, these findings strongly suggest that the AKM Lab‐01 and GCE combination may exert synergistic anti‐obesity effects via a gut microbiota–host metabolic axis, supporting its potential as a novel synbiotic strategy for obesity management.

## Graphical abstract

Combined administration of pasteurized Akkermansia muciniphila (AKM Lab‐01) and Garcinia cambogia extract (GCE) alleviates high‐fat‐diet‐induced obesity in mice by reducing adiposity, metabolic disturbances, and adipose inflammation, while remodeling gut microbiota and adipose transcriptome, revealing a gut‐host metabolic axis that underpins the synergistic anti‐obesity efficacy.

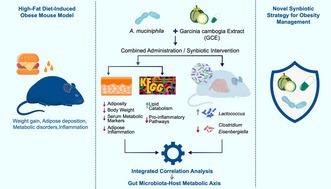

## 1 Introduction

Obesity is a multifaceted metabolic disorder marked by pathological accumulation of adipose tissue, which significantly increases health risks. Global data from the World Health Organization (WHO) in 2022 indicate that approximately one in eight people worldwide are affected by obesity, reflecting its escalating burden as a public health concern (WHO 2025; Zhao 2013). Beyond its direct physiological consequences, obesity serves as a major predisposing factor for several non‐communicable diseases, including type 2 diabetes, cardiovascular diseases, neurological disorders, and gastrointestinal conditions (Khan et al. 2021). An elevated body mass index (BMI) beyond the optimal range is estimated to contribute to approximately 3.7 million deaths annually, highlighting the urgent need for effective and sustainable therapeutic strategies (Collaborators, G. B. D. R. F 2024). Although traditional interventions—such as dietary regulation, physical activity, and pharmacotherapy—remain essential, growing attention is being directed toward novel biological targets for obesity prevention and management, particularly the gut microbiota (Bluher et al. 2023).

The human gut microbiota comprises trillions of microorganisms primarily residing in the large intestine and functions as a dynamic and complex ecosystem that plays a critical role in host metabolism, energy homeostasis, and immune modulation (Cani et al. 2019; Sender et al. 2016; Zhang et al. 2019). This microbial community not only facilitates the digestion of complex dietary components but also generates bioactive metabolites that modulate host physiology. In obesity, gut microbiota dysbiosis is frequently observed, typically characterized by decreased microbial diversity and an elevated ratio of Firmicutes to Bacteroidetes (F/B ratio). This compositional shift has been linked to enhanced energy extraction from the diet, persistent low‐grade inflammation, and excessive adipose tissue deposition (Liu et al. 2021; Van Hul and Cani 2023; Zhao 2013). Importantly, the gut microbiota produces metabolites such as short‐chain fatty acids (SCFAs), secondary bile acids, and indole derivatives, which influence host metabolism, appetite regulation, and inflammation (Lin et al. 2022; Van Hul and Cani 2023).

Among the beneficial microbes, *Akkermansia muciniphila* ( *A. muciniphila* , AKK) has shown promise in improving gut barrier function and insulin sensitivity. Since it was first isolated from the feces of a healthy adult and described as an intestinal mucin‐degrading bacterium in 2004 (Derrien et al. 2004), AKK has gained considerable attention in the past two decades. AKK is regarded as a next‐generation probiotic because it has exhibited numerous positive effects in human health and multiple diseases, including modulating host immunity (Pena‐Cearra et al. 2024), enhancing the intestinal barrier integrity (Reunanen et al. 2015), regulating gut‐brain axis (Fang et al. 2021), and promoting metabolic health in diabetes, obesity, and fatty liver disease (Dao et al. 2016). There is accumulating evidence of the correlations between AKK and obesity. Both human and animal studies confirmed that the abundance of AKK in the gut was negatively correlated with obesity (Dao et al. 2016; Schneeberger et al. 2015), and the supplementation of AKK to the obese individuals and high‐fat diet‐induced obese mice significantly improved obesity and corresponding metabolic states (Depommier et al. 2019; Xiao et al. 2024), indicating the benefits of AKK in managing obesity. Moreover, multiple factors are involved in the mechanism of AKK improving obesity. An 84 kDa protein, named P9 and derived from AKK, was identified to interact with intercellular adhesion molecule 2 (ICAM‐2), induce GLP‐1 secretion and brown adipose tissue thermogenesis, thus ameliorating obesity in mice (Yoon et al. 2021). As an important functional component, membrane protein Amuc_1100 of AKK was also indicated to improve obesity by activating the AC3/PKA/HSL pathway and facilitating lipolysis (Zheng et al. 2023). Another study observed that AKK‐derived extracellular vesicles improved glucose tolerance mainly through enhancing tight junction function (Chelakkot et al. 2018; Sun et al. 2025). In our prior work, we isolated and characterized a novel strain of *Akkermansia muciniphila* , termed AKM Lab‐01 (Huang et al. 2026). Administration of this strain via oral gavage for 4 weeks markedly suppressed high‐fat diet‐induced obesity in mice, including reduced weight gain, improved glucose homeostasis, decreased circulating lipid levels, and enhanced insulin sensitivity. A subsequent 90‐day dietary toxicity study additionally verified the safety of AKM Lab‐01 in mice. Currently, two clinical trials are ongoing to evaluate the efficacy of this strain in patients with hypercholesterolemia (NCT06974266) and obesity (NCT07331974).

Notably, accumulating evidence has confirmed that pasteurized AKK possesses stronger anti‐obesity effects than viable strains, which are mediated by multiple synergistic mechanisms (Depommier et al. 2019, 2020; Plovier et al. 2017). Pasteurization can not only maintain the biological activity of core functional proteins such as Amuc_1100 but also further amplify their functions by facilitating the release and surface exposure of these active substances while eliminating adverse metabolic disturbances caused by live bacteria. Moreover, inactivated AKK exerts more potent effects in ameliorating obesity, insulin resistance, and glucose intolerance, repairing intestinal barrier integrity, relieving endotoxemia, and accelerating energy expenditure in pre‐clinic and clinic studies (Depommier et al. 2019, 2020; Plovier et al. 2017). In addition, AKK is an obligate anaerobe that is extremely sensitive to oxygen. Its survival and intestinal colonization capacity are easily disturbed by intestinal microenvironmental factors including pH value and interspecific bacterial competition, thereby resulting in unstable in vivo efficacy. More importantly, viable bacterial strains may bring potential infection risks to immunocompromised subjects and other high‐risk populations. Collectively, the excellent stability and favorable biosafety profile make pasteurized AKK a more promising candidate for future clinical translational research.

Plant extracts act as natural modulators of the gut microbiota, regulating community structure and function. For instance, they can promote butyrate‐producing bacteria to enhance butyrate production and modify host antioxidant capacity (Perez‐Burillo et al. 2020). Recent studies indicate that plant extracts, including those from traditional Chinese medicine, alleviate various metabolic diseases (e.g., type 2 diabetes, obesity, and cardiovascular diseases). Their therapeutic benefits may be partly mediated by exerting beneficial biogenic effects on *A. muciniphila* , thereby modulating its abundance, distribution, and metabolic activity to enhance probiotic function (Bu et al. 2020). Specific examples include rhubarb supplementation, which increases *A. muciniphila* levels and protects against diet‐induced obesity and diabetes in mice (Regnier et al. 2020), and the synbiotic combination of quercetin with *A. muciniphila* , which ameliorates early obesity and NAFLD by reshaping the gut microbiota and modulating bile acid metabolism (Juarez‐Fernandez et al. 2021). These findings collectively suggest that combining *A. muciniphila* with medicinal plant extracts holds promise for synergistic disease therapy. Notably, in current studies, the primary form of *A. muciniphila* used in such combinations is the live bacterium (Juarez‐Fernandez et al. 2021). The therapeutic potential of its pasteurized form in combination with plant extracts remains to be investigated.

*Garcinia cambogia*, commonly referred to as Malabar tamarind, is a tropical fruit originating from Southeast Asia and Southwestern India. It has long been used in both traditional cooking and folk medicine in these regions (Semwal et al. 2015). Hydroxycitric acid (HCA), which is abundant in the fruit rind, has been found to be a major factor in garcinia's weight loss properties (Noreen et al. 2023). HCA exerts its effects on reducing fat synthesis and suppressing appetite through inhibition of ATP citrate lyase, a key enzyme involved in de novo lipogenesis. (Li et al. 2017). Recent studies have also explored the lipid‐lowering effects of *G. cambogia* extract (GCE). For example, supplementation with GCE in mice resulted in lower levels of triglyceride‐rich lipoproteins in plasma and upregulation of lipoprotein receptor expression in the liver. (Hanse et al. 2023). Furthermore, animal studies have also demonstrated that combining GCE with specific probiotic strains can produce synergistic anti‐obesity effects. For example, co‐administration of *Lactobacillus plantarum* HAC03 and *G. cambogia* extract in a diet‐induced obesity mouse model resulted in significant reductions in weight gain, adipocyte size, and lipogenic gene expression than either treatment alone. Additionally, this combination also modulated gut microbiota composition and metabolic pathways, suggesting a therapeutic potential of dual‐intervention techniques and pointing to a microbiota‐mediated mechanism of action (Kang et al. 2023). However, the combined efficacy of *A. muciniphila* and *G. cambogia* on obesity and their impact on the gut microbiota‐metabolism relationship remain unclear. Notably, although numerous studies have confirmed the anti‐obesity potential of *Garcinia cambogia*, its associated adverse risks cannot be ignored, including hepatotoxicity (Stevens et al. 2005), gastrointestinal disorders (Fassina et al. 2015) and myocarditis (Marquez et al. 2012). Accordingly, combined administration of *Garcinia cambogia* with viable bacteria may further elevate the incidence of side effects. Given the excellent biosafety and robust anti‐obesity efficacy of pasteurized AKK, the combined application of pasteurized AKK and Garcinia cambogia is considered a promising therapeutic strategy for obesity intervention.

Based on these findings and the future potential application, we hypothesized that the combination of *G. cambogia* extract and pasteurized *A. muciniphila* AKM Lab‐01 might exert enhanced synergistic effects in the treatment of obesity. In this study, we evaluated the co‐treatment in a diet‐induced obese mouse model. The combined intervention led to further improvements in key serum biochemical parameters related to obesity‐associated metabolic markers, as well as in fat pad mass and adipose tissue histology. To gain deeper mechanistic insights, we performed transcriptomic analysis of adipose tissue, which revealed an enhancement of lipid metabolism‐related pathways and a concomitant suppression of obesity‐related inflammatory signatures. Metagenomic analysis further indicated shifts in gut microbial composition, characterized by an increased abundance of *Lactococcus* and decreased abundance of *Clostridium* and *Eisenbergiella*. Additionally, correlation analysis between the transcriptome and metagenome suggested that changes in the abundance of *Lactococcus*, *Clostridium*, and *Eisenbergiella* were associated with alterations in adipose tissue signaling pathways. Moreover, employing a 3 T3‐L1 adipocyte in vitro model, we confirmed that *Lactococcus* plays a potential role in regulating lipid metabolism and inflammation. Collectively, these results imply that the combined intervention produces synergistic therapeutic effects against obesity, potentially mediated by microbiota‐associated regulated mechanisms.

## 2 Materials and Methods

### 2.1 Material Preparation

Pasteurized *A. muciniphila* AKM Lab‐01 powder was purchased from Moon Biotech Co. Ltd. (Catalog number: 0100005592). The suspension of *A. muciniphila* AKM Lab‐01 was prepared immediately before use by adding 9 mL of saline to 1 g of pasteurized powder and vortexing at 2500 rpm for 30 s, yielding a final concentration of 1 × 10^11^ TFU/mL. GCE was obtained from Changsha Huir Biological‐Tech Co. LTD (Batch number: 244‐23052904). The solution of GCE with a final concentration of 200 mg/mL was prepared immediately before use by dissolving 0.8 g of extract in 4 mL of saline.

### 2.2 Animal Experiments

Male C57BL/6J mice, aged 5 to 6 weeks, were obtained from GemPharmatech Inc. The animals were housed under specific pathogenfree (SPF) c‐onditions, with ambient temperature maintained at 24°C ± 2°C, relative humidity at 50% ± 10%, and a 12‐h light/dark cycle. Except during designated fasting periods, all mice had free access to food and water. All animal procedures were conducted in accordance with protocols approved by the Institutional Animal Care and Use Committee of Moon Biotech Co. Ltd. (IACUC approval number: IACUC202411‐6O), following the guidelines set forth in the Guide for the Care and Use of Laboratory Animals. The animal experiment was conducted in accordance with our previous study (Huang et al. 2026), including the administration dosage of AKM Lab‐01, treatment duration, and physiological as well as biochemical examinations. This study was designed as a therapeutic model to evaluate the anti‐obesity effects of AKM Lab‐01, GCE, or their combination. Following a one‐week acclimation period, a total of 35 mice were placed on a high‐fat diet (HFD) for 4 weeks. The HFD provided 5.2 kcal/g, with 20% of energy from protein, 60% from fat, and 20% from carbohydrate (Diet D12492; Research Diets Inc., New Brunswick, NJ, USA). Then, mice with a weight of 30 ± 2 g (*n* = 20) were divided into four groups (*n* = 5 per group)—the HFD group, the AKM Lab‐01 group, the GCE group, and the AKM Lab‐01 + GCE group, in which mice were administered by gavage with saline (0.2 mL, per mouse), AKM Lab‐01 (0.2 mL, 2 × 10^10^ TFU per mouse), GCE (1000 mg/kg), and AKM Lab‐01 plus GCE at the same dose, respectively. The administration lasted for 8 weeks with a continuous high‐fat diet. One week before the end of the experiment, glucose tolerance tests were performed. On day 56, all mice were fasted overnight for 12 h, after which fasting blood glucose levels were measured. On day 57, after collecting blood from the ocular venous plexus, euthanasia was performed via cervical dislocation following deep anesthesia induction with 4%–5% isoflurane (R510‐22‐10, Jinan Ante Biochemical Pharmaceutical Co. Ltd.) in oxygen (200–400 mL/min) delivered via an inhalation anesthesia system (CL‐1000‐S4, Shanghai Yuyan Instruments Co. Ltd.) within an induction chamber, and tissues were collected. Notably, overdose anesthetic (e.g., pentobarbital) will be preferentially adopted as the primary euthanasia procedure, as this approach alleviates animal distress and fully complies with AVMA‐recommended standards. After sample collection, serum biochemical parameters, including total cholesterol (TCHO), triacylglycerol (TG), low‐density lipoprotein (LDL), high‐density lipoprotein (HDL), alanine aminotransferase (ALT), aspartate aminotransferase (AST), blood urea nitrogen (BUN), and creatinine (CRE) were tested by Wuhan Servicebio Technology Co. Ltd. using an automatic biochemical analyzer (Chemray 240, Rayto).

### 2.3 Oral Glucose Tolerance Test

One week prior to the end of the experiment, glucose tolerance tests were performed. Following a 12 h fasting period, mice were weighed and then given an oral glucose solution at a dose of 2 g per kg of body weight. Blood was sampled from the tail tip at 0, 30, 60, 90, and 120 min post‐administration for serum glucose measurement. Glucose concentrations were determined using a glucometer with glucose test strips.

### 2.4 Histological Staning and Analysis

Liver and adipose tissue specimens were fixed in paraformaldehyde, sectioned, and stained with hematoxylin and eosin (H&E). Histopathological examination was performed by Ningbo Yangming Medical Inspection Laboratory Co. Ltd. The tissue was photographed at 100× magnification.

### 2.5 Transcriptomic Analysis

Total RNA extraction was carried out by Meige Genetics BioTech Co. Ltd. (Guangzhou, China). RNA degradation and potential contamination were assessed via electrophoresis on 1% agarose gels. RNA concentration was determined using Qubit 4.0 (Thermo Fisher Scientific, Waltham, USA) and Nanodrop One (Thermo Fisher Scientific, MA, USA). RNA integrity was accurately assessed using the Agilent 4200 system (Agilent Technologies, Waldbronn, Germany). Sequencing libraries were prepared using ALFA‐SEQ RNA Library Prep Kit (Cat. NRI002E‐03). The library was sequenced on an Illumina NovaSeq 6000 platform, generating 150 bp paired‐end reads. Raw reads were filtered for quality using fastp (version 0.20.0). The resulting clean reads were mapped to the GRCm39 reference genome with STAR (version 2.7.4a). Gene expression was quantified by StringTie (version 2.2.1) as transcripts per million (TPM). Differential expression analysis was subsequently performed using the limma package in R (version 3.56.2).

### 2.6 Metagenomic Analysis

Bacterial genomic DNA was extracted by Guangdong Magigene Biotechnology Co. Ltd. (Guangzhou, China) using commercial kits. DNA integrity and purity were assessed via 1% agarose gels. DNA concentration and purity were measured using Qubit 3.0 (Thermo Fisher Scientific, Waltham, USA) and Nanodrop One (Thermo Fisher Scientific, Waltham, USA). Sequencing libraries were constructed using the ALFA‐SEQ DNA Library Prep Kit. The library quality was assessed on the Qubit 4.0 Fluorometer (Life Technologies, Grand Island, NY) and Qsep400 High‐Throughput Nucleic Acid Protein Analysis system (Houze Biological Technology Co, Hangzhou, China). Finally, the libraries were sequenced on an Illumina NovaSeq 6000 platform, generating 150 bp paired‐end reads. Raw sequencing reads were filtered for quality using fastp (version 0.20.0). Host reads were removed with Kraken2 (version 2.1.3) and Bracken (version 2.9) by aligning to the GRCm39 reference genome, and relative species abundance was calculated for each sample. Functional abundance was estimated using HUMAnN (version 3.0.0.alpha.4). The differential analysis among groups was performed using Kruskal‐Wallis test followed by Dunn's multiple comparisons test. The principal co‐ordinates analysis (PCoA) was performed by vegan package of R. To explore the functional implications of differentially abundant genera, we extracted their Level 4 Enzyme Commission abundances, ranked the features by log_2_ fold change, and conducted Gene Set Enrichment Analysis (GSEA) to identify enriched KEGG pathways. Spearman correlation analysis between species relative abundance and gene expression was performed using custom R scripts.

### 2.7 Bacteria Culture and Supernatant Preparation

*Lactococcus cremoris* strains Lc‐12 and Lc‐13, isolated from healthy human feces, were cultured in a self‐optimized MM01 liquid medium (pH 6.3–6.5) containing per liter: 15 g peptone, 20 g glucose, 15 g yeast extract, 1 g cysteine, 5 g sodium acetate, 4 g sodium citrate, 2 g dipotassium phosphate, 0.1 g magnesium sulfate, 0.05 g manganese sulfate, and 1 g Tween 80. The cultures were incubated for 48 h. Bacterial culture supernatant was then obtained by centrifuging the bacterial pellet at 4500 rpm, followed by filtration through a 0.22‐μm filter membrane to remove residual bacteria.

### 2.8 Cell Culture and Induction of 3 T3‐L1 Adipocytes Differentiation and Inflammation

3 T3‐L1 preadipocytes were cultured in Dulbecco's modified Eagle medium (DMEM, Gibco, REFC1995500BT, China) supplemented with 1% penicillin–streptomycin (Gibco, 15140‐122, China) and 10% fetal bovine serum (FBS; Pricella, 164210‐50, China) at 37°C in a 5% CO_2_ incubator. Cells were passaged upon reaching 80%–90% confluence.

For induction of adipocytes differentiation, 3 T3‐L1 preadipocytes at the logarithmic growth phase were seeded into 24well plates at a density of 5 × 10^4^ cells/cm^2^ and cultured in 10% FBS—DMEM until reaching complete confluence (designated as day 0). After two additional days of culture (day 2) to achieve contact inhibition, the medium was replaced with differentiation induction medium (DM), which consisted of DMEM supplemented with 10% FBS, 0.5 mmol/L 3‐isobutyl‐1‐methylxanthine (IBMX; Macklin, I811775, China), 1.0 μmol/L dexamethasone (Sigma‐Aldrich, D54370, USA), and 10 mg/L insulin (Beyotime, P3376, China). After 2 days of incubation (day 2), DM was aspirated and replaced with differentiation maintenance medium (MM) composed of DMEM containing 10% FBS and 10 mg/L insulin. The cells were cultured for an additional 2 days (until day 4), after which the medium was changed to basal maintenance medium (DMEM with 10% FBS), refreshed every 2 days.

For induction of inflammatory adipocytes, after 3 T3‐L1 cells had been induced to differentiate into adipocytes for 10 days (day 10), the medium was replaced with inflammatory induction medium (InfM). InfM consisted of DMEM supplemented with 10% FBS and a cocktail of pro‐inflammatory cytokines, including 20 ng/mL IL‐6 (Yeasen, Cat. No. 90146ES05, China), 20 ng/mL IL‐1β (Yeasen, 90140ES05, China), 20 ng/mL TNF‐α (Yeasen, 90621ES08, China), and 20 ng/mL CXCL1 (Yeasen, 90921ES08, China). Cells were then cultured for an additional 2 days.

### 2.9 Oil Red O Staining

Adipocyte differentiation was assessed by Oil Red O staining. Differentiated 3 T3‐L1 cells were gently washed twice with PBS and then fixed with 4% paraformaldehyde (Servicebio, G1101, China) for 20–30 min at room temperature. After fixation, cells were washed three times with PBS and stained with Oil Red O working solution for 20–30 min at room temperature using an Oil Red O Staining Kit (Beyotime, C0157S, China). Subsequently, cells were quickly rinsed with 60% isopropanol to remove background staining. Images were captured under an inverted microscope, and intracellular lipid droplets in mature adipocytes appeared orange‐red. The intensity of Oil Red O staining was quantified using ImageJ software.

### 2.10 RNA Extraction and RT‐qPCR Analysis

Total RNA was extracted from 3 T3‐L1 cells using the MiniBEST Universal RNA Extraction Kit (Takara, 9767, China). cDNA was synthesized from the extracted RNA using the PrimeScript RT Master Mix (Takara, RR036A, China). Quantitative real‐time PCR was performed on an ABI PRISM 7500 Fast system using TB Green Premix Ex Taq II (Takara, RR820A, China). The relative expression levels of target genes were calculated using the 2^−ΔΔCt^ method, with *Gapdh* as the internal control. The primer sequences were as follows: Table 1.

**TABLE 1:** qPCR primer pairs for the target gene.

Gene	Forward primer (5′ → 3′)	Reverse primer (5′ → 3′)
*Fasn*	TCCTGGAACGAGAACACGATCT	GAGACGTGTCACTCCTGGACTTG
*Scd1*	TTCTTGCGATACACTCTGGTGC	CGGGATTGAATGTTCTTGTCGT
*Pparg*	GTACTGTCGGTTTCAGAAGTGCC	ATCTCCGCCAACAGCTTCTCCT
*Il1b*	TGGACCTTCCAGGATGAGGACA	GTTCATCTCGGAGCCTGTAGTG
*Il6*	TACCACTTCACAAGTCGGAGGC	CTGCAAGTGCATCATCGTTGTTC
*Tnfa*	GTAGCCCACGTCGTAGCAAA	TTGAGATCCATGCCGTTGGC
*Gapdh*	CATCACTGCCACCCAGAAGACTG	ATGCCAGTGAGCTTCCCGTTCAG

### 2.11 Statistical Analysis

Data were presented as mean ± standard deviation (S.D.). Sample sizes and statistical methods were provided in Figureure or table legends. GraphPad software (version 10.2.3) or Provantis 10.5.0.4 were used for statistical analyses. For physiological and biochemical parameters, normality was first assessed using the Shapiro–Wilk test within each group. For datasets in which all groups passed the normality test (*p* > 0.05), one‐way *ANOVA* followed by Dunnett's multiple comparisons test was applied. For endpoints where any group deviated significantly from normality (*p* < 0.05), the non‐parametric Kruskal–Wallis test with Dunn's multiple comparison test was used instead. For time‐course data, two‐way ANOVA with appropriate post hoc correction was performed. Given the inherent complexity and compositional nature of metagenomic and transcriptomic data, all comparisons for these datasets were uniformly conducted using the Kruskal–Wallis test with Dunn's post hoc test to ensure statistical robustness. Significance levels are indicated as: ns, not significant; *p < 0.05; **p < 0.01; ***p < 0.001; ****p < 0.0001.

## 3 Results

### 3.1 Combination of AKM Lab‐01 and GCE Improved Obesity‐Related Symptoms in Diet‐Induced Obese Mice

**FIGURE 1:**
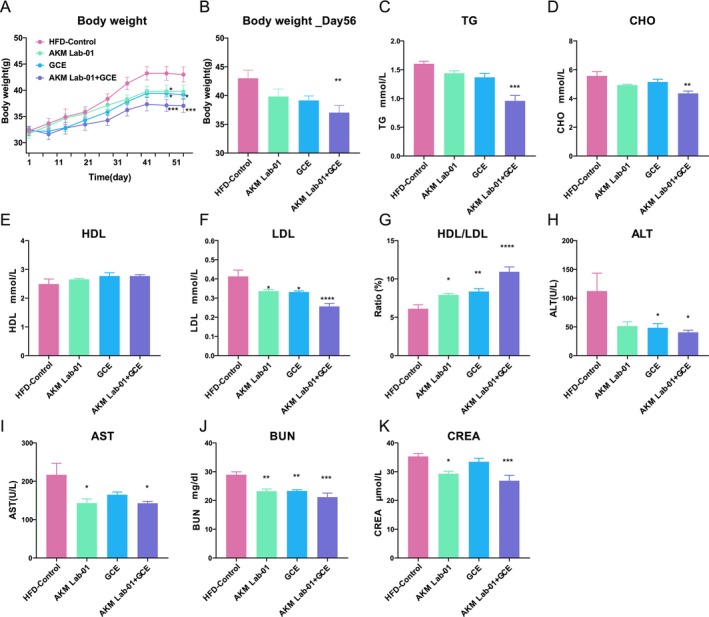
Combination of AKM Lab‐01 and *Garcinia cambogia* improved obesity‐related symptoms in HFD‐induced obese mice. (A) Body weight curve of HFD‐induced obese mice with AKM Lab‐01, GCE or combination. Mice were fed with HFD for 4 weeks to induce obesity. Subsequently, the mice were randomly divided into four groups and daily gavaged with saline, AKM Lab‐01 (0.2 mL, 2 × 10^10^ TFU per mouse), GCE (1000 mg/kg), or combination of AKM Lab‐01 and GCE, respectively. (B) Body weight on Day 56. (C–K) Serum chemistry parameters were measured by using an automatic biochemical analyzer. TG (C), TCHO (D), HDL (E), LDL (F), HDL/LDL ratio (G), ALT (H), AST (I), BUN (J), and CRE (K). Data are presented as mean ± SD. Statistical analysis was performed using two‐way *ANOVA* for the line chart or one‐way *ANOVA* for the bar chart combined with Dunnett's multiple comparisons test compared with HFD‐control. For (C, D, I), the non‐parametric Kruskal‐Wallis test was applied because the data were not normally distributed. ns, not significant, not shown; *p < 0.05, **p < 0.01, ***p < 0.001, ****p < 0.0001.

To evaluate the effects of combining pasteurized *A. muciniphila* AKM Lab‐01 and *G. cambogia* extract (GCE) on obesity and related metabolic disorders, we employed a high‐fat diet (HFD)‐induced obesity model for evaluating the therapeutic effect. Key parameters including body weight and serum biochemical profiles were assessed during 8 weeks of intervention. As previously reported (Huang et al. 2026), both AKM Lab‐01 and GCE monotherapies led to reductions in body weight (Figure 1A,B). Notably, the combination treatment resulted in a more pronounced reduction (13.86%, vs. HFD‐Control) in body weight compared to either intervention alone (Figure 1A,B). In the assessment of glucose homeostasis, AKM Lab‐01 alone reduced the area under the curve (AUC) in an oral glucose tolerance test (OGTT, Figure S1A–C) and lowered fasting blood glucose levels (Figure S1D), which is consistent with previous observations. However, the combination of AKM Lab‐01 and GCE did not lead to further improvement in glucose levels (Figure S1). In the blood lipid assay, the combination treatment with AKM Lab‐01 and GCE resulted in a more pronounced reduction in serum TG (40.27%, vs. HFD‐Control) and TCHO (21.80%, vs. HFD‐Control) levels compared to the monotherapy groups (Figure 1C,D). No significant differences were observed in HDL levels across the four groups (Figure 1E). In contrast, LDL was 37.93% decrease following co‐treatment (Figure 1F). Consequently, the HDL/LDL ratio also exhibited a notable reduction (Figure 1G). Furthermore, the combination of AKM Lab‐01 and GCE significantly ameliorated the dysfunction parameters of both liver and kidney, as reflected by marked improvement in the serum levels of ALT and AST for liver function (Figure 1H,I), and BUN and CRE for renal function (Figure 1J,K). Collectively, these findings demonstrate that the combination of AKM Lab‐01 and GCE ameliorates diet‐induced obesity and its associated lipid metabolism in the HFD‐induced obese mouse model.

### 3.2 Combination of AKM Lab‐01 and GCE Regulated Lipid Metabolism in HFD‐Induced Obese Mice

**FIGURE 2:**
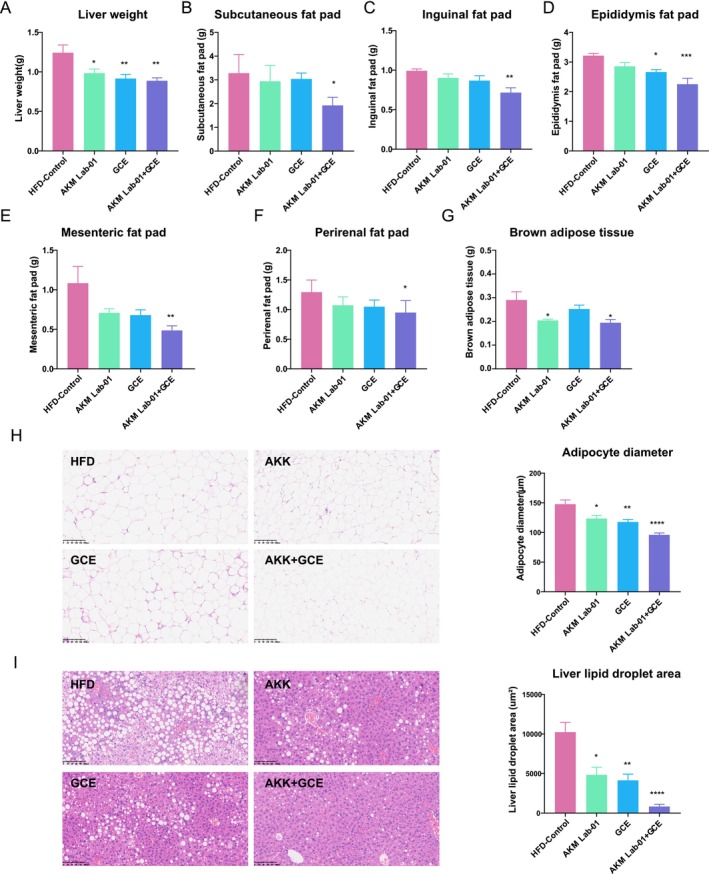
Combination of AKM Lab‐01 and *G. cambogia* attenuates adipose tissue pathology in HFD‐induced obese mice. (A) Weight of liver tissue. (B–G) Weight of various adipose tissues. Subcutaneous fat pad (B), Inguinal fat pad (C), Epididymis fat pad (D), Mesenteric fat pad (E), Perirenal fat pad (F), Brown adipose tissue (G). (H) Adipose tissue histology and adipocyte size analysis. Representative histological images of epididymal fat pads from the indicated groups and the corresponding quantitative analysis of adipocyte size. (I) Liver tissue histology and lipid droplet area analysis. Representative histological images of liver tissue of indicated groups and the corresponding quantitative analysis of lipid droplet area. Data are presented as mean ± SD. Statistical analysis was performed by one‐way *ANOVA* for the bar chart combined with Dunnett's multiple comparisons test, which compared with HFD‐control. For (B), the non‐parametric Kruskal‐Wallis test was applied because the data were not normally distributed. ns: Not significant, not shown; *p < 0.05, **p < 0.01, ***p < 0.001, ****p < 0.0001.

We next examined the impact of combination therapy on adiposity and hepatic steatosis in diet‐induced obese mice. Quantitative dissection showed that AKM Lab‐01 plus GCE produced a greater reduction in liver mass than either agent alone (Figure 2A). This superiority extended to all major fat depots: subcutaneous, inguinal, epididymal, mesenteric and perirenal pads were consistently lighter in the combination group (Figure 2B–F), as was brown adipose tissue (Figure 2G). Histologically, the decrease in adipose mass corresponded to smaller epididymal adipocyte cross‐sectional areas (Figure 2H) and, in the liver, to a marked contraction of lipid droplet size (Figure 2I). Collectively, these data indicate that combined AKM Lab‐01 and GCE reverses lipid accumulation and adipose tissue pathology in HFD‐fed mice.

### 3.3 Combination of AKM Lab‐01 and GCE Promotes Lipid Metabolism and Inhibits the Inflammation Pathway in Adipose Tissue

**FIGURE 3:**
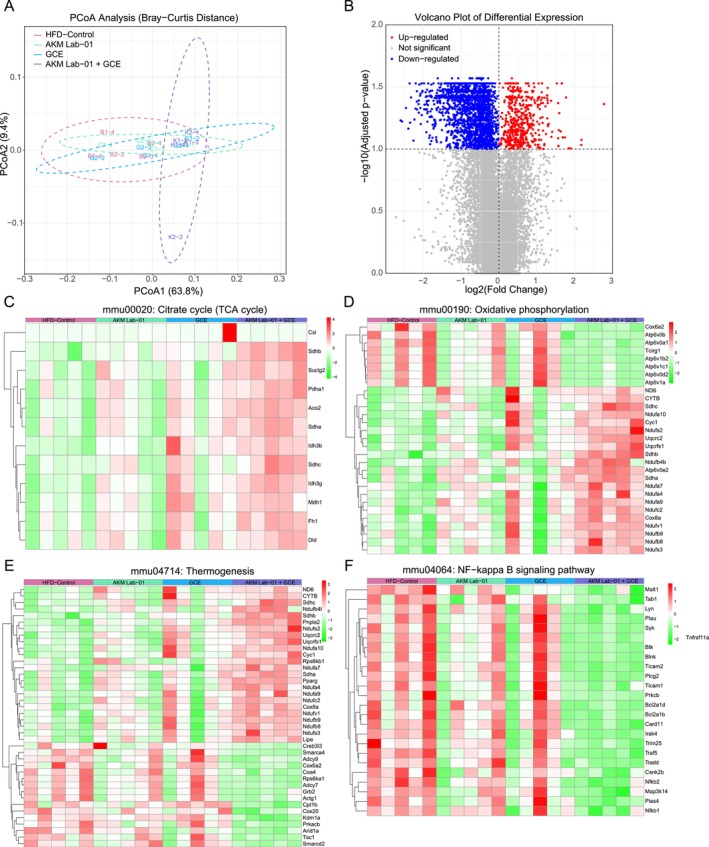
Combination of AKM Lab‐01 and *Garcinia cambogia* promotes lipid metabolism and suppresses inflammatory pathways in adipose tissue. (A) PCoA of the gene's expression in epididymal fat pads from the indicated group in HFD‐induced obese mice. (B) Volcano plot shows the different expressed genes compared between HFD control group and AKM Lab‐01 + GCE group. Red points represent the up‐regulated gene, while blue shows the down‐regulated (*p* < 0.05 and FDR < 0.1). (C–F) Heatmap of lipid metabolism‐related KEGG pathways enriched in differentially expressed genes between HFD control and AKM Lab‐01 + GCE groups. Heatmaps displaying expression patterns of key pathways: Citrate cycle (C), Oxidative phosphorylation (D), Thermogenesis (E), and NF‐κB signaling pathway (F).

To further elucidate the mechanism by which the combined administration of AKM Lab‐01 and GCE ameliorates obesity‐related symptoms and modulates lipid metabolism, we performed transcriptome profiling (RNA‐seq) on epididymal adipose tissue. Principal coordinate analysis revealed distinct clustering of gene expression profiles across experimental groups (Figure 3A). Differential expression analysis identified 2527 differentially expressed genes (DEGs) between the combination group and HFD‐induced control group (*p* < 0.05 and FDR < 0.1), comprising 496 up‐regulated and 2031 down‐regulated genes (Table S1 and Figure 3B).

To elucidate the biological processes modulated by the combination of *Akkermansia muciniphila* AKM Lab‐01 and GCE, we performed KEGG pathway enrichment analysis. DEGs were significantly enriched in 83 KEGG pathways (*p* < 0.05 and FDR < 0.1, Table S2). Given that fatty acids are oxidized in mitochondria to acetyl‐CoA, which enters the TCA cycle and subsequently drives oxidative phosphorylation to produce ATP for cellular energy supply (Serra et al. 2013), we focused on pathways related to energy metabolism. Enrichment analysis indicated that DEGs were primarily associated with obesity and lipid metabolism regulation, including the citrate cycle (TCA cycle), oxidative phosphorylation, and thermogenesis (Figure 3C–E). Notably, most genes in the citrate cycle pathway were upregulated following combination treatment with AKM Lab‐01 and *Garcinia cambogia* (Figure 3C). Key enzymes such as *Pdha1*, *Aco2*, *Idh3b*, *Idh3g*, *Sdha*, and *Sdhc*, which are critical for enhancing TCA cycle flux, were among those elevated. Furthermore, multiple mitochondrial genes, including *Ndufs2*, *Ndufa10*, *Uqcrc2*, and *Cox8a*, were upregulated in both oxidative phosphorylation and thermogenesis pathways (Figure 3D–E), indicating enhanced adipose tissue metabolism via improved mitochondrial function. In addition, the combination treatment markedly downregulated most genes in the NF‐κB signaling pathway in adipose tissue (Figure 3F). These results suggest that the combination of AKM Lab‐01 and *GCE* promotes lipid metabolism by enhancing mitochondrial function and suppressing inflammatory signaling in adipose tissue.

### 3.4 Combination of *Akkermansia muciniphila* AKM Lab‐01 and GCE Remodeled Gut Microbiota in HFD‐Induced Obese Mice

**FIGURE 4:**
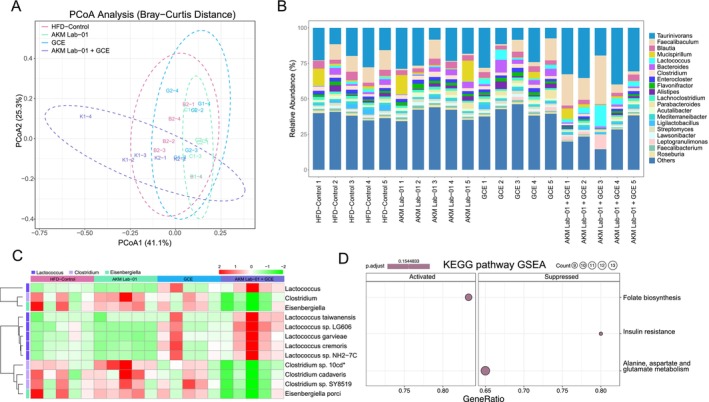
The combination of AKM Lab‐01 and *G. cambogia* remodels the gut microbiota in HFD‐induced obese mice. (A) PCoA based on Bray‐Curtis distance at the species level (*p* = 0.001). (B) Relative abundance of the predominant bacterial genera. (C) Heatmap of the most significantly altered genera and species‐level composition within these genera across groups. (D) GSEA of *Lactococcus* (*p* < 0.05, FDR < 0.2).

To further elucidate the systemic effects of the combination treatment, we conducted a metagenomic analysis of the gut microbiota in an HFD‐induced mouse model. Alpha diversity, as measured by observed species (Figure S2A) and the Shannon index (Figure S2B), did not differ significantly among the groups. However, principal coordinates analysis (PCoA) revealed distinct separation in microbial community structure between groups (Figure 4A). The top 20 most abundant genera were visualized using a stacked column chart (Figure 4B). A significant difference in the abundance of gut microbiota among the groups was assessed using the Kruskal–Wallis test, followed by Dunn's multiple comparisons test (*p* < 0.05, Table S3). Among the predominant genera, the relative abundance of *Lactococcus* was notably increased in the group treated with the combination of AKM Lab‐01 and *Garcinia cambogia*, whereas the abundances of *Clostridium* and *Eisenbergiella* were significantly reduced (Figure 4C). Consistent with these genus‐level changes, corresponding species—*Lactococcus cremoris*, *Clostridium cadaveris* , and *Eisenbergiella porci*—also showed significant alterations at the species level in the combination treatment group compared with the HFD‐control group (Figure 4C).

To infer the potential functional contributions of these differentially abundant genera, we conducted Gene Set Enrichment Analysis (GSEA) based on KEGG pathways using enzyme commission (EC) level 4 annotations derived from the metagenomic data. For *Lactococcus*, pathways related to folate biosynthesis, insulin resistance, and alanine, aspartate and glutamate metabolism were significantly enriched (Figure 4D; *p* < 0.05, FDR < 0.2). Of particular interest, the enrichment of insulin resistance—a hallmark of obesity‐associated metabolic dysfunction (Barber et al. 2021; Zhao et al. 2023)‐ was suppressed in the *Lactococcus*‐enriched group, suggesting that *Lactococcus* may contribute to metabolic improvement. As no synergistic reduction in glucose concentrations was observed following combined treatment with AKM Lab‐01 and GCE (Figure S1), additional correlation analyses were conducted to explore associations between *Lactococcus* abundance and glucose parameters. Pooled analysis across all experimental groups revealed a moderate yet statistically non‐significant negative correlation. Of note, the correlative tendency became even weaker upon restricting the analysis to the AKM monotherapy and combination‐treatment cohorts (Figure S3). These data suggest that *Lactococcus* partially modulates insulin secretion; nevertheless, the modest magnitude of this regulatory effect fails to elicit significant declines in circulating glucose levels. In contrast to *Lactococcus*, GSEA for *Clostridium* and *Eisenbergiella* did not identify any pathways meeting the significance threshold (FDR > 0.2, Supplementary Table S4). This indicates that neither genus shows potential functional predictability for metabolism‐related pathways based on the current dataset, and thus their biological roles require further systematic investigation. Collectively, these results suggest that the combination of AKM Lab 01 and Garcinia cambogia may potentially remodel the gut microbiota in HFD‐fed mice and could contribute to a metabolic profile conducive to the improvement of lipid homeostasis.

### 3.5 Correlations Between Gut Microbiota and Adipose Tissue Metabolic Pathways

**FIGURE 5:**
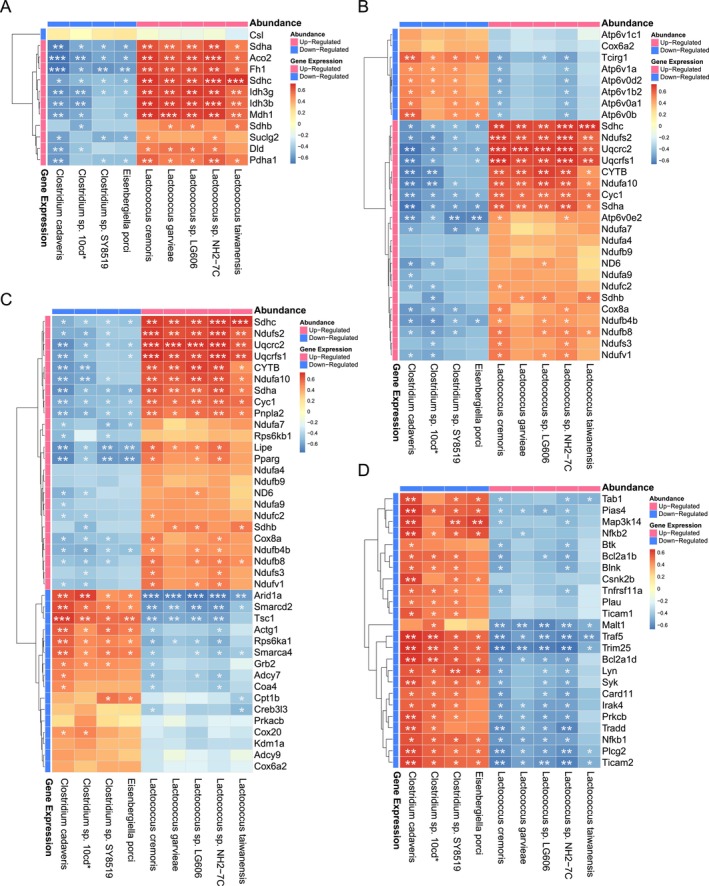
Correlation between gut microbiota and adipose tissue metabolic reprogramming. (A–D) Spearman correlation analysis between significantly altered bacterial species and differentially expressed genes (DEGs) in key pathways. Heatmaps depict correlation coefficients for the Citrate cycle (A), Oxidative phosphorylation (B), Thermogenesis (C), and the NF‐κB signaling pathway (D). Coefficients range from −0.6 to 0.6. Significant correlations are marked with asterisks: *p* < 0.05, *p < 0.01, **p < 0.001. Species with increased abundance are colored pink, decreased blue; up‐regulated genes are red, down‐regulated green.

To investigate the link between altered gut microbiota and adipose tissue metabolism, we performed Spearman correlation analyses focusing on differential species (selected from differential abundant genera) and significantly altered metabolic pathways in adipose tissue (Table S3 and Figure 5). A positive correlation was found between the abundance of *Lactococcus*‐associated species and the upregulation of TCA cycle genes, in contrast to the negative correlations identified for species from *Clostridium* and *Eisenbergiella* (Figure 5A). Similar trends were noted for the Oxidative Phosphorylation and Thermogenesis pathways (Figure 5B,C). Notably, the prevalence of mitochondrial genes among these upregulated transcripts suggests that the relevant bacterial species may conceivably regulate adipose tissue metabolism by influencing mitochondrial functions. Furthermore, the abundance of *Lactococcus* species was inversely correlated with the expression of NF‐κB pathway genes, whereas *Clostridium* and *Eisenbergiella* species showed a positive correlation (Figure 5D), implying a potential involvement of these microbes in regulating adipose tissue inflammation.

### 3.6 Effect of *Lactococcus cremoris* on Adipocyte Differentiation and Inflammation

**FIGURE 6:**
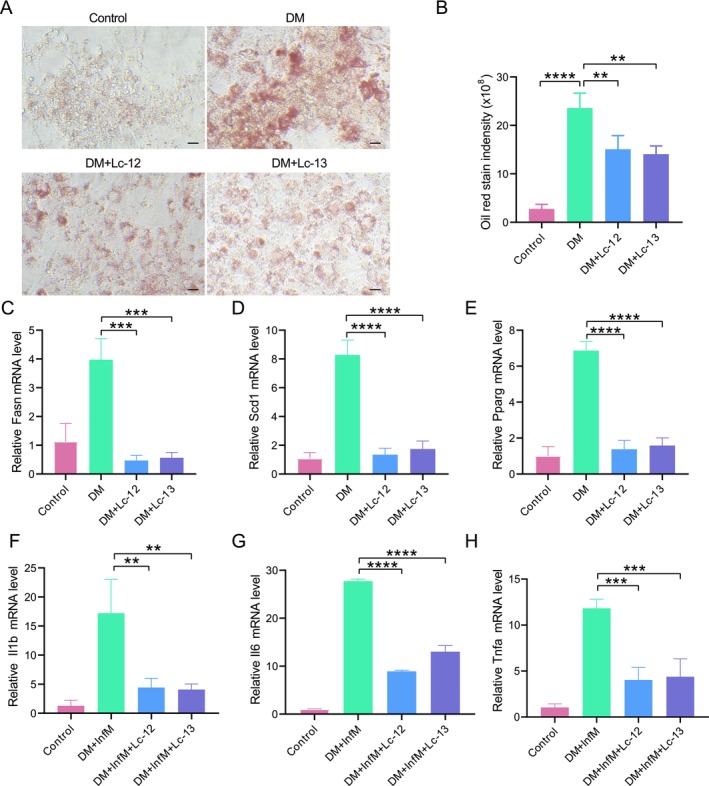
Effect of *Lactococcus cremoris* on adipocyte differentiation and inflammation. (A) Representative images of Oil Red O staining of 3 T3‐L1 cells induced to differentiate into adipocytes under the indicated treatments. Scale bar = 50 μm. (B) Quantification of Oil Red O staining intensity in each group. (C–E) Transcript levels of lipid metabolism‐related factors in differentiated 3 T3‐L1 adipocytes, including (C) fatty acid synthase (*Fas*); (D) stearoyl‐CoA desaturase 1 (*Scd1*); and (E) peroxisome proliferator‐activated receptor‐gamma (*Pparg*), determined by RT‐qPCR. (F–H) Expression of pro‐inflammatory cytokines in differentiated 3 T3‐L1 adipocytes under inflammatory induction, including (F) *Il1b*; (G) *Il6*; and (H) *Tnfa*, determined by RT‐qPCR. Data are presented as mean ± SD. Statistical analysis was performed by one‐way *ANOVA* for the bar chart combined with Dunnett's multiple comparisons test, which compared with DM or DM + InfM group. ns: Not significant, not shown; **p < 0.01, ***p < 0.001, ****p < 0.0001.

To further investigate the potential of *Lactococcus* species in improving adipocyte metabolism and mitigating inflammation, we employed a 3 T3‐L1 adipocyte in vitro model to examine the effects of *Lactococcus cremoris* on adipocyte differentiation. The impact of *Lactococcus cremoris* strains Lc‐12 and Lc‐13 on 3 T3‐L1 adipocyte differentiation was assessed by Oil Red O staining (Figure 6A). Compared with cells treated with differentiation medium (DM) alone, supplementation with the culture supernatant of either Lc‐12 or Lc‐13 led to a marked reduction in cellular lipid accumulation (Figure 6B). We further evaluated the influence of *Lactococcus cremoris* on the expression of lipid accumulation factors in differentiated 3T3L1‐adipocytes (Figure 6C–E). The mRNA levels of *Fasn*, *Scd1*, and *Pparg* were significantly decreased following Lc‐12 or Lc‐13 treatment (Figure 6C–E), an effect similar to that reported for another *Lactococcus* species, *Lactococcus chungangensis* CAU 28 (Zhang et al. 2020). In addition, we used a cytokine‐induced inflammatory 3 T3‐L1 adipocyte model (Cartier et al. 2026) to assess the anti‐inflammatory effect of *L. cremoris* . Treatment with inflammatory cytokine‐containing medium (InfM) significantly increased the expression of pro‐inflammatory genes such as *Il1b*, *Il6*, and *Tnfa*; conversely, Lc‐12 or Lc‐13 treatment markedly suppressed the expression of these inflammatory genes (Figure 6F–H). Collectively, these data demonstrate the potential of *Lactococcus cremoris* to regulate adipogenic metabolism and mitigate inflammation.

## 4 Discussion

This study confirmed that the combined intervention of pasteurized *Akkermansia muciniphila* AKM Lab‐01 and GCE exhibits a significant synergistic anti‐obesity effect in HFD‐induced obese mouse models. Specifically, the combination showed greater efficacy in reducing body weight gain, improving dyslipidemia, and decreasing visceral fat accumulation compared with either AKM Lab‐01 or GCE alone. Further mechanistic investigations revealed that the exertion of this synergistic effect relies on the remodeling of intestinal flora structure (e.g., enrichment of *Lactococcus* spp. and reduction of pro‐inflammatory related genera) and the coordinated regulation of metabolic‐inflammatory pathways in adipose tissue, providing new experimental evidence and mechanistic insights for the development of nutritional intervention strategies for obesity.

*Akkermansia muciniphila* is recognized as a next‐generation probiotic with considerable promise for managing metabolic diseases (Jian et al. 2023; Vallianou et al. 2023). Its efficacy against obesity‐related disorders has been confirmed for both live and pasteurized forms in preclinical and clinical studies (Depommier et al. 2019; Plovier et al. 2017). Notably, the pasteurized form has been reported to improve several metabolic parameters, such as insulin sensitivity, blood lipid profiles, and body weight. Recently, we have validated that our proprietary strain, *Akkermansia muciniphila* AKM Lab‐01, is safe and effective against obesity in both live and pasteurized forms (Huang et al. 2026). As a postbiotic, pasteurized AKK preserves its bioactive constituents while ensuring greater stability and eliminating the safety concerns typically associated with live bacteria (Cuevas‐Gonzalez et al. 2020), underscoring its potential for food and medical applications.

Separately, *Garcinia cambogia* extract, whose active component is hydroxycitric acid (HCA), has well‐documented anti‐obesity and gut microbiota‐modulating effects (Dong et al. 2023; Kim et al. 2008). Furthermore, its combination with live probiotics has demonstrated robust synergistic efficacy against obesity (Heo et al. 2016; Kang et al. 2023).

Despite the individual benefits of pasteurized AKK and GCE, whether their combination yields a synergistic anti‐obesity effect remains unexplored. To develop a superior anti‐obesity therapy, we evaluated the combined effect of pasteurized AKM Lab‐01 and GCE in HFD‐induced obese mice. As expected, pasteurized AKM Lab‐01 or GCE alone reduced body weight by approximately 7% and 8%, respectively, compared to the HFD control. Notably, the combination of both supplements led to a further reduction of more than 13.8%. Consistently, key metabolic parameters were significantly improved in the combination group, which exhibited reductions of 40.3% in TG, 21.8% in TC, and 37.9% in LDL levels versus the HFD control. These findings reveal that the combination of pasteurized AKM Lab‐01 and GCE represents a potentially promising nutritional intervention strategy against obesity.

*G. cambogia* suppresses appetite and decreases fat synthesis by blocking ATP citrate lyase, a critical enzyme involved in de novo lipogenesis (Li et al. 2017). Previous research has shown that *L. plantarum* HAC03 combined with *G. cambogia* significantly reduces the mass of subcutaneous (SAT), mesenteric (MAT), and brown (BAT) adipose tissues, but not epididymal adipose tissue (EAT) (Kang et al. 2023). In the present study, the combination of pasteurized AKM Lab‐01 and GCE extended this effect, significantly reducing not only SAT, MAT, and BAT but also EAT, as well as inguinal (IAT) and perirenal (PAT) adipose tissues. Liver fat accumulation was also improved. These findings indicate that the pasteurized AKM Lab‐01/GCE combination has a broader impact and a potential unique role in regulating lipid metabolism across diverse adipose depots. Furthermore, pasteurized AKM Lab‐01 not only retains functional probiotic components (e.g., the Amuc_1100 protein) but also enhances safety and stability, emphasizing the promise of this combinatorial strategy for obesity intervention.

To investigate the molecular mechanisms by which the combination therapy affects adipose tissue, we conducted transcriptome profiling in HFD‐induced mice. Principal coordinate analysis (PCoA) revealed that the combined intervention significantly reshaped the global gene expression profile. A total of 2527 differentially expressed genes (DEGs) were identified (496 up‐regulated and 2031 down‐regulated) compared to the HFD control (*p* < 0.05, FDR < 0.1). KEGG enrichment analysis linked these DEGs to key metabolic pathways, including the citrate cycle (TCA cycle), oxidative phosphorylation, and thermogenesis. Notably, the expression of pivotal TCA cycle enzymes (e.g., *Pdha1*, *Aco2*, *Idh3b*, *Idh3g*, *Sdha*, *Sdhc*) was significantly up‐regulated, suggesting an enhanced TCA cycle flux (Cianciulli Sesso et al. 2021; Wu et al. 2018) (Xiao et al. 2021). Similarly, up‐regulation of mitochondrial genes involved in oxidative phosphorylation and thermogenesis (e.g., *Ndufs2*, *Ndufa10*, *Uqcrc2*, *Cox8a*) indicated an improvement in mitochondrial function (Sakaguchi et al. 2022; Schwach et al. 2024). In contrast, genes involved in the NF‐κB pathway, which is associated with inflammation and known to be activated in obesity (Luo and Lin 2021; Rohm et al. 2022), were down‐regulated following the combination treatment. Although monotherapy with either pasteurized AKM lab‐01 or GCE also up‐regulated these genes, the combination therapy produced a markedly stronger effect, underscoring its synergistic action. This systematic analysis suggests that the combination of AKM Lab‐01 and GCE may exert synergistic effects on modulating lipid metabolism, which is potentially associated with improved mitochondrial function and attenuated inflammatory signaling in adipose tissue.

Gut microbiota is essential for human health. Conversely, its disruption, known as dysbiosis, has been linked to a wide spectrum of diseases, including gastrointestinal and metabolic disorders, autoimmune diseases, as well as neurological and psychiatric conditions (Fujimoto et al. 2022; Liu et al. 2019; Tu et al. 2023). AKK can ameliorate diseases by modulating the composition and abundance of the gut microbiota. For instance, through its mucolytic activity, AKK fosters the colonization of the butyrate‐producing commensal *Anaerostipes caccae* (Chia et al. 2018). Furthermore, AKK exhibits a syntrophic relationship with *Faecalibacterium prausnitzii* , another key commensal bacterium, as both co‐occur in the mucosal layer. Notably, the abundances of these two syntrophic species are concurrently reduced in inflammatory bowel disease (Lopez‐Siles et al. 2018), underscoring their potential role in maintaining gut homeostasis. Previous studies have demonstrated that the combination of GCE and probiotics can alleviate weight gain and adiposity in high‐fat diet‐fed mice, partly through differential modulation of the gut microbiota (Heo et al. 2016). However, whether AKK and GCE collaboratively enhance anti‐obesity effects via gut microbiota modulation remains to be elucidated. In this study, we employed metagenomic sequencing to elucidate the composition of the gut microbiota in HFD‐induced obese mice following the indicated treatments. Our analysis revealed that while the alpha diversity of the gut microbiota did not differ significantly among the groups, the pasteurized AKM lab‐01 and GCE combination intervention group exhibited a considerable shift in microbial community structure compared to the other groups, as assessed by principal coordinates analysis (PCoA). Moreover, we found that the abundance of *Lactococcus* was significantly increased in the combination group, whereas that of *Clostridium* and *Eisenbergiella* decreased among the highly abundant species. These microbial shifts align well with existing literature. The elevated abundance of *Clostridium* under HFD conditions and the increase in specific *Lactococcus* species following probiotic or GCE supplementation (Heo et al. 2016) directly support the observed changes. Furthermore, the reduction in *Eisenbergiella* is also documented in other anti‐obesity interventions, such as indole‐3‐carbinol treatment, which conversely promotes *Lactococcus* (Mao, Gao, et al. 2024). Notably, species within the genus *Lactococcus* have been implicated in the regulation of obesity. For instance, the combination of *Lactococcus lactis subsp. lactis* LL‐1 and *Lacticaseibacillus paracasei* LP‐16 was reported to modulate the gut microbiota and metabolites, thereby exerting anti‐obesity and hypolipidemic effects in mice (Gao et al. 2025). Similarly, *Lactococcus chungangensis* CAU 28 was shown to alleviate diet‐induced obesity and modulate adipose tissue metabolism both in vitro and in high‐fat diet‐fed mice (Zhang et al. 2020). In addition, *Lactococcus lactis subsp. cremoris* has been found to protect against metabolic alterations induced by a Western‐style diet (Naudin et al. 2020). In our study, multiple *Lactococcus* species, including *Lactococcus cremoris*, *Lactococcus garvieae* , and *Lactococcus taiwanensis*, were significantly enriched in the combination group, further supporting the potential role of this genus in modulating metabolic health during obesity. Notably, enrichment analysis via GSEA revealed a significant contribution of *Lactococcus* to the suppression of the insulin resistance pathway under the combination intervention. In contrast, although the abundances of *Clostridium* and *Eisenbergiella* were markedly decreased in the combined intervention group, the absence of significant GSEA pathways precludes any functional inference from the current data, which merits further experimental verification in future research.

Although we observed that pasteurized AKM lab‐01 and GCE combination modulated obesity‐related pathways in adipose tissue, the role of gut microbiota shifts in this metabolic regulation remained unclear. Advancing beyond prior probiotic‐GCE research (Kang et al. 2023), our study further employed a multimodal ‘omics’ approach—integrating transcriptomics with metagenomics—to provide a more comprehensive, mechanistic insight into the AKK‐GCE synergy. With this approach, we strikingly found that species of *Lactococcus* were positively correlated with the upregulation of genes in the TCA cycle, oxidative phosphorylation, and thermogenesis pathways, while being negatively correlated with inflammatory pathway genes. In contrast, *Clostridium* and *Eisenbergiella* species exhibited an opposite correlation pattern. These results imply that beneficial remodeling of gut microbiota may potentially participate in alleviating obesity during AKK and GCE co‐treatment.

Previous studies (Gao et al. 2025; Naudin et al. 2020; Zhang et al. 2020) together with our multimodal omics approach have linked *Lactococcus* to the regulation of lipid metabolism and inflammation. Using a 3T3L1 adipocyte differentiation model, we found‐ that *Lactococcus cremoris* supernatant alone was sufficient to prevent lipid accumulation, suggesting that metabolites produced by this bacterium regulate adipocyte lipid metabolism. Fatty acid synthase (*Fasn*) is a key enzyme in the de novo synthesis of long‐chain saturated fatty acids (Gunenc et al. 2022), while stearoyl‐CoA desaturase 1 (*Scd1*) catalyzes the conversion of saturated to monounsaturated fatty acids (Flowers and Ntambi 2008). We observed that *Lactococcus cremoris* supernatant significantly decreased the expression of both *Fasn* and *Scd1*, implying a role in limiting lipid accumulation. Furthermore, activation of peroxisome proliferator‐activated receptor gamma (*Pparg*) promotes adipocyte differentiation and upregulates fatty acid synthesisrelate‐d genes including *Fasn* (Choi et al. 2020; Zhang et al. 2020). Treatment with *Lactococcus cremoris* supernatant markedly reduced *Pparg* mRNA levels. Collectively, these results indicate that *Lactococcus cremoris* may improve adipocyte lipid metabolism by downregulating key lipogenic genes. Adipose tissue expansion in obesity triggers adipocyte hypertrophy, hypoxia, ER stress, and cell death, leading to M1 macrophage polarization and subsequent overproduction of proinflammatory cytokines such as TN‐F‐α, IL‐1β, and IL6, along with dysregulated ad‐ipokine secretion (Ajoolabady et al. 2023) (Mirabelli et al. 2024) (Maliniak et al. 2021) (Caslin et al. 2020). In a pro‐inflammatory cytokineinduced‐3T3L1 adipocyte inflammation model,‐*Lactococcus cremoris* supernatant significantly suppressed the inflammatory response by reducing the expression of *Il1b*, *Il6*, and *Tnfa*. These findings suggest that *Lactococcus cremoris* may serve as one of the key mediators contributing to the anti‐obesity effects observed during co‐treatment with AKM Lab‐01 and GCE.

It is important to note several limitations in our study. While our study establishes that the pasteurized *A. muciniphila* (AKM lab‐01) and GCE combination alleviates obesity through gut microbiota remodeling, the precise molecular mechanisms warrant deeper mechanistic dissection. Although correlation analyses and in vitro biological validation have linked specific taxa (e.g., *Lactococcus*) to improved adipose tissue metabolism and inflammation, these observations do not establish causality. A key limitation is the lack of metabolomic data to functionally link microbial restructuring to host metabolic pathways. It is well recognized that gut microbes influence systemic physiology largely through metabolites such as short‐chain fatty acids, tryptophan derivatives, and bile acids (Deng et al. 2023; Mao, Paerhati, et al. 2024; Wang et al. 2024). Therefore, future studies should integrate metabolomics with metagenomics to identify and validate microbiota‐derived metabolites that mediate the anti‐obesity effects. Furthermore, the functional role of key correlated species (e.g., *Lactococcus*) could be examined using gnotobiotic models, monocolonization, or targeted metabolite supplementation to delineate their direct crosstalk with host metabolic and inflammatory pathways. Ultimately, the downstream targets of identified metabolites should be validated through genetic o‐r pharmacological interventions in vitro and in vivo to clarify the complete signaling axis. Notably, although functional annotation revealed that microbial shifts induced by the combination treatment (primarily involving *Lastococcus*) were associated with suppressed insulin resistance, the addition of GCE did not further improve blood glucose levels beyond pasteurized AKM Lab‐01 alone. Further correlation analysis revealed a moderate but nonsignificant negative correlation b‐etween *Lactococcus* abundance and glucose parameters. To explain this discrepancy in glucose metabolism, we propose the following interpretation. GSEA reflects pathwaylevel predictions rather than direct functional meas‐urements, thus, the *Lactococcus*associated suppressio‐n of insulin resistance pathways is an *in silico* signal that may not translate into improved glucose tolerance when other factors change, such as in the combination treatment group. *Lactococcus* may act as an active mediator rather than a mere passenger bacterium. However, the combination treatment likely introduced microbial interactions that dampened this signal, leading to the loss of correlation between *Lactococcus* and glucose in that group. Additionally, other bacterial taxa or metabolic cross‐feeding effects could have counteracted the predicted pathway activity. Therefore, this discrepancy is not a true contradiction but rather a reminder that pathway enrichment reflects statistical association, not mechanistic causality. In addition, the absence of additional glucose improvement in the combination group is not explained by GCE dose insufficiency, as our dose (1000 mg/kg) is comparable to a reported effective dose (Dong et al. 2023). Rather, microbial interactions in the combination group may have dampened the glucose‐lowering signal observed in the GCE‐alone group, and a longer intervention might be needed for adipose‐tissue benefits to translate into improved glucose tolerance (Dong et al. 2023). Further studies are needed to dissect these distinct regulatory mechanisms. Addressing these points will strengthen the mechanistic foundation for the translational development of pasteurized AKM lab‐01 and GCE as a microbiota‐targeted therapy for obesity.

When considering the use of probiotic‐based interventions, safety remains the foremost concern—particularly for next‐generation probiotics such as *A. muciniphila* and *Faecalibacterium prausnitzii* , which have only recently been introduced into clinical practice. Although the safety of AKK has been established in recent preclinical (Druart et al. 2021; Huang et al. 2026) and clinical (Depommier et al. 2019; Lee et al. 2024) studies, the safety profile of its combination with GCE still requires thorough investigation. *G. cambogia* is widely included in weight‐loss supplements; however, excessive or prolonged intake has been linked to digestive discomfort and an elevated risk of liver injury, primarily due to high levels of its active component HCA (Noreen et al. 2023). A comprehensive case series and literature review by Crescioli et al. (2018) documented a total of 66 patients with adverse events following G. cambogia consumption, including 50 cases of acute liver injury, 8 requiring liver transplantation, and 2 deaths. Most of these serious events occurred at high doses (> 2000 mg/day), with prolonged use, or in multi‐ingredient formulations (e.g., Hydroxycut). Another rare but notable adverse event is ocular complications (angle closure, myopic shift, uveal effusion), reported in a patient taking more than twice the recommended dose (Cho et al. 2020). Importantly, dosage is closely tied to safety outcomes. A dose–response meta‐analysis of eight randomized controlled trials (total *n* = 530) by Golzarand et al. (2020) found that *G. cambogia* at daily doses ranging from 166 to 4667 mg significantly reduced body weight, BMI, body fat percentage, and waist circumference, with no serious adverse events reported. Nevertheless, the optimal daily dose for supplementation remains to be fully established in future studies. In preclinical studies, the maximum reported doses of GCE were 5000 mg/kg for short‐term and 2500 mg/kg for long‐term evaluations in rats, with no mortality or serious adverse events observed (Marquez et al. 2012). Similarly, studies in obese mouse models using doses of 1000 mg/kg (Hanse et al. 2023) or even 10,000 mg/kg (Kim et al. 2008) also reported no fatalities or severe adverse effects. In our study, we used GCE at 1000 mg/kg and observed no mortality or serious adverse events, supporting the suitability of this dosage for our combination strategy. Nevertheless, further studies should systematically evaluate the safety of GCE when co‐administered with pasteurized AKM Lab‐01, with particular attention to its mechanism of action and optimal dosage. Considering the potential risks associated with *G. cambogia*, we will also intend to explore other dietary supplements with reported anti‐obesity properties, such as conjugated linoleic acid, glucomannan, green coffee extract, green tea extract, and white kidney bean extract, etc. (Bonetti et al. 2022), as alternative candidates for combined use with *Akkermansia*‐based formulations in obesity management.

Notably, as an exploratory pilot study with a limited sample size (*n* = 5 per group), our investigation may be underpowered to detect small‐to‐moderate effect sizes. Consequently, non‐significant findings should be interpreted with caution, and independent validation in larger cohorts is warranted. Nevertheless, the large effect sizes observed for all primary endpoints, together with post hoc power exceeding 0.85, support the robustness of our main conclusions. In addition, we acknowledge the lack of a pair‐fed control and daily food intake measurements. Thus, we cannot rule out that some metabolic benefits (e.g., weight loss) may partly result from reduced caloric intake. However, our study was a therapeutic model in already obese mice, and the combination treatment enhanced mitochondrial lipid metabolism and reduced adipose inflammation, which are not typical features of simple caloric restriction. This suggests that the treatment improves metabolism beyond any potential reduction in food intake. This limitation does not affect our main conclusion that the combination of GCE and AKK is associated with improvements in metabolic syndrome alongside alterations in gut microbiota.

Taken together, our study demonstrates that the combined intervention of pasteurized AKM lab‐01 and GCE exerts synergistic anti‐obesity effects, primarily mediated through the remodeling of the gut microbiota and its associated metabolic functions. These findings lend support to the development of AKK‐based synbiotic strategies, leveraging natural products, as a novel therapeutic approach against obesity.

## 5 Conclusion

In this study, we confirm that co‐administration of pasteurized *Akkermansia muciniphila* (AKM lab 01) and GCE exerts synergistic anti‐obesity effects in a high‐fat diet murine model, with superior improvements in body weight, serum lipid profiles, and hepatic/renal function compared to either monotherapy. This combination effectively reduces adiposity and hepatic steatosis, thereby restoring systemic lipid homeostasis. Transcriptomic analysis reveals that the synergistic effect is mediated by the upregulation of mitochondrial pathways (including the TCA cycle, oxidative phosphorylation, and thermogenesis) in adipose tissue and the suppression of the NF‐κB inflammatory response. Concurrently, metagenomic profiling shows that the treatment remodels the gut microbiota, enriching *Lactococcus* and depleting *Clostridium* and *Eisenbergiella*, with functional enrichment suggesting *Lactococcus* contributes to metabolic improvement via modulation of insulin resistance‐related pathways. Correlation analysis further links these microbial shifts to adipose tissue metabolic and transcriptional changes, implying a microbiota–adipose axis as the potential mediator of the combination's systemic benefits. Using a 3 T3‐L1 adipocyte model, we confirmed that *Lactococcus* plays a potential role in regulating lipid metabolism and inflammation. Future clinical trials are now warranted to validate these findings in humans and to further elucidate the microbiota‐adipose signaling axis, which will be critical for translating these preclinical results into potential therapeutic strategies for obesity and related metabolic disorders. Our results strongly suggest that AKM Lab‐01 combined with GCE alleviates obesity‐related metabolic dysfunction, pointing toward a mechanism involving enhanced mitochondrial lipid metabolism, attenuated adipose tissue inflammation, and favorably reshaping the gut microbiota. These findings highlight the therapeutic potential of this probiotic–botanical combination as a multimodal strategy for managing obesity and its metabolic complications.

## Author Contributions

**Wenbin Xue:** methodology, data curation, visualization, conceptualization, formal analysis. **Baojia Huang:** conceptualization, methodology, data curation, visualization, writing – original draft, formal analysis. **Zhou Lan:** visualization, formal analysis. **Zilun Pu:** methodology, software, formal analysis, writing – original draft, data curation. **Sherlyn Sze Ning Koay:** writing – original draft, methodology. **Yibo Xian:** writing – review and editing, supervision, conceptualization. **Zhipeng Chen:** conceptualization, writing – original draft, methodology, formal analysis, data curation, visualization. **Lihong Tai:** methodology, formal analysis, data curation. **Amanda Juan Chen:** conceptualization, writing – review and editing, funding acquisition, supervision. **Ping Kong:** methodology, data curation, formal analysis. **Yingying Zhao:** methodology, formal analysis, data curation. **Yalin Zhou:** writing – original draft, methodology.

## Funding

This work was funded by the Special Projects in Key Fields of Ordinary Universities of Guangdong Province (grant numbers: 2024ZDZX2077).

## Disclosure

*Author Approval and Responsibility Statement*: All authors have read and approved the final version of the manuscript. Amanda Juan Chen and Yibo Xian had full access to all of the data in this study and take complete responsibility for the integrity of the data and the accuracy of the data analysis.

## Ethics Statement

The animal experiment was approved by the Institutional Animal Care and Use Committee of Centre Testing International (Guangzhou) Co. Ltd., in compliance with the Guide for the Care and Use of Laboratory Animals (IACUC Number: IACUC202411‐6O, approval date: 2024.12.05).

## Conflicts of Interest

The authors declare no conflicts of interest.

## Supporting information


**Figure S1:** Combination of AKM Lab‐01 and Garcinia cambogia does not significantly reduce blood glucose levels in HFD‐Induced Obese mice. (A) Curve of OGTT. (B) AUC of OGTT. (C) Blood Glucose Levels at 2 h Post‐Treatment in the OGTT. D. Fasting blood glucose level on Day56. Data are presented as mean ± SD. Statistical analysis was performed by two‐way *ANOVA* for the line chart or one‐way *ANOVA* for the bar chart combined with Dunnett's multiple comparisons test, which compared with HFD‐control. ns: not significant, not showed.
**Figure S2:** Alpha diversity of the gut microbiota across the indicated group in HFD‐Induced Obese mice. Observed species (A), Shannon index (B)
**Figure S3:** Correlation analysis between *Lactococcus* abundance and glucose parameters. A. Pooled analysis across all experimental groups. B. Analysis between the AKM monotherapy and combination‐treatment group.

**Table S1:** Comparative analysis of differentially expressed genes (DEGs) between the HFD control and AKM Lab‐01 + GCE combination groups.

**Table S2:** KEGG pathway enrichment analysis of differentially expressed genes (DEGs) between the HFD control and AKM Lab‐01 + GCE combination groups.

**Table S3:** Analysis of Relative Gut Microbiota Abundance.

**Table S4:** Functional enrichment analysis of pathways associated with differential genera.

## Data Availability

The metagenomic and transcriptomic data have been deposited in the NCBI database under BioProject (Accession number: PRJNA1401928). The authors confirm that the data supporting the findings of this study are available within the article and/or its Supporting Information.
