# Supplementary material for: Synergistic Anti‐Obesity Effect of Akkermansia muciniphila AKM Lab‐01 and Garcinia cambogia Extract via Gut Microbiota Remodeling in Diet‐Induced Obese Mice
Source: Food Sci Nutr. 2026 Jul 28;14(8):e72140. doi: 10.1002/fsn3.72140 (PMC13415990; doi:10.1002/fsn3.72140)
Supplement: Supplementary file 1 — Figure S1: Combination of AKM Lab‐01 and Garcinia cambogia does not significantly reduce blood glucose levels in HFD‐Induced Obese mice. (A) Curve of OGTT. (B) AUC of OGTT. (C) Blood Glucose Levels at 2 h Post‐Treatment in the OGTT. D. Fasting blood glucose level on Day56. Data are presented as mean ± SD. Statistical analysis was performed by two‐way ANOVA for the line chart or one‐way ANOVA for the bar chart combined with Dunnett's multiple comparisons test, which compared with HFD‐control. ns: not significant, not showed. Figure S2: Alpha diversity of the gut microbiota across the indicated group in HFD‐Induced Obese mice. Observed species (A), Shannon index (B) Figure S3: Correlation analysis between Lactococcus abundance and glucose parameters. A. Pooled analysis across all experimental groups. B. Analysis between the AKM monotherapy and combination‐treatment group. [file FSN3-14-e72140-s001.docx]

# **Supplementary Materials for**

**Synergistic Anti-Obesity Effect of *Akkermansia muciniphila* AKM Lab-01 and *Garcinia cambogia* Extract via Gut Microbiota Remodeling in Diet-Induced Obese Mice**

**Authors and Affiliations**

Baojia Huang^1†^, Zhipeng Chen^1†^, Wenbin Xue^1^, Zilun Pu^1^, Yalin Zhou^2,3^, Sherlyn Sze Ning Koay^2,3^, Ping Kong^1^, Yingying Zhao^1^, Lihong Tai^1^, Zhou Lan^1^, Yibo Xian^1^**^*^**, Amanda Juan Chen^1^**^*^**

^1^ Moon (Guangzhou) Biotech Co. Ltd., Huangpu District, Guangzhou, Guangdong, 510530, China

2 Department of Chemical Engineering, Tsinghua University, Beijing 100084, China

3 Key Lab of Industrial Biocatalysis, Ministry of Education, Tsinghua University, Beijing 100084, China

*Author to whom correspondence should be addressed.

† These authors contributed equally to this work

This file includes

**Figure S1**. Combination of AKM Lab-01 and *Garcinia cambogia* does not significantly reduce blood glucose levels in HFD-Induced Obese mice

**Figure S2**. Alpha diversity of the gut microbiota across the indicated group in HFD-Induced Obese mice.

**Figure S3**. Correlation analysis between *Lactococcus* abundance and glucose parameters.


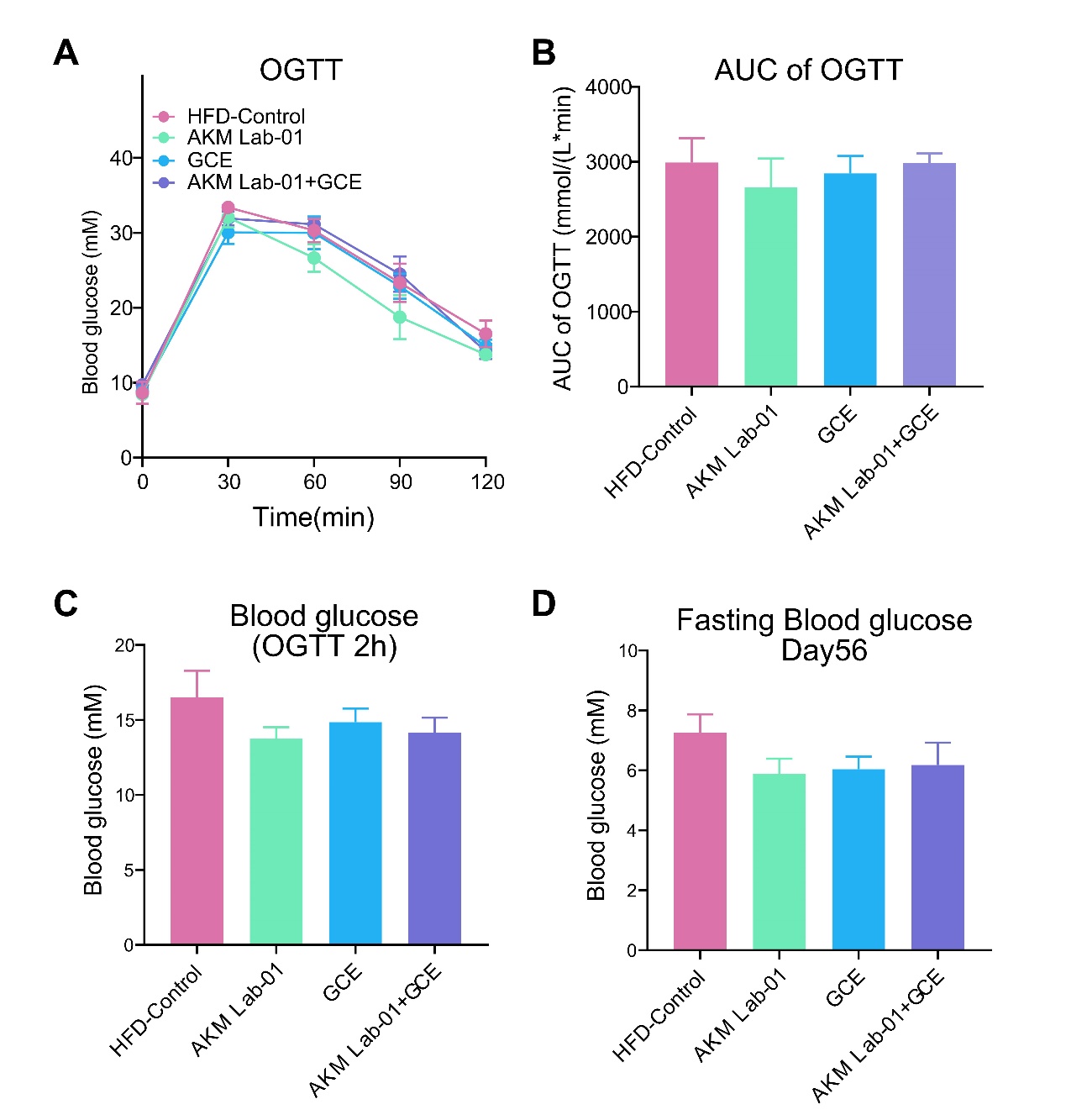


**Figure S1. Combination of AKM Lab-01 and Garcinia cambogia does not significantly reduce blood glucose levels in HFD-Induced Obese mice. A.** Curve of OGTT. **B.** AUC of OGTT. **C.** Blood Glucose Levels at 2 Hours Post-Treatment in the OGTT. **D**. Fasting blood glucose level on Day56. Data are presented as mean ± SD. Statistical analysis was performed by two-way *ANOVA* for the line chart or one-way *ANOVA* for the bar chart combined with Dunnett’s multiple comparisons test, which compared with HFD-control. ns: not significant, not showed.


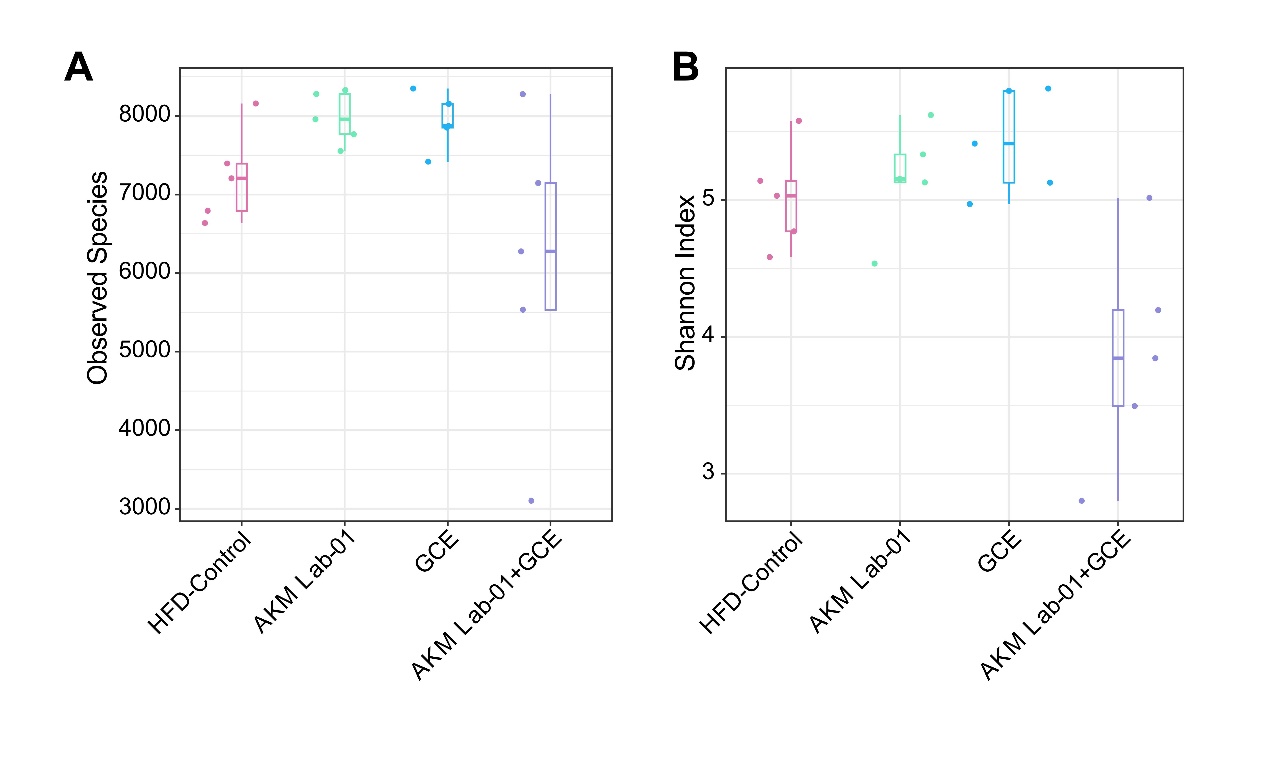


**Figure S2. Alpha diversity of the gut microbiota across the indicated group in HFD-Induced Obese mice.** Observed species (**A**), Shannon index (**B**)


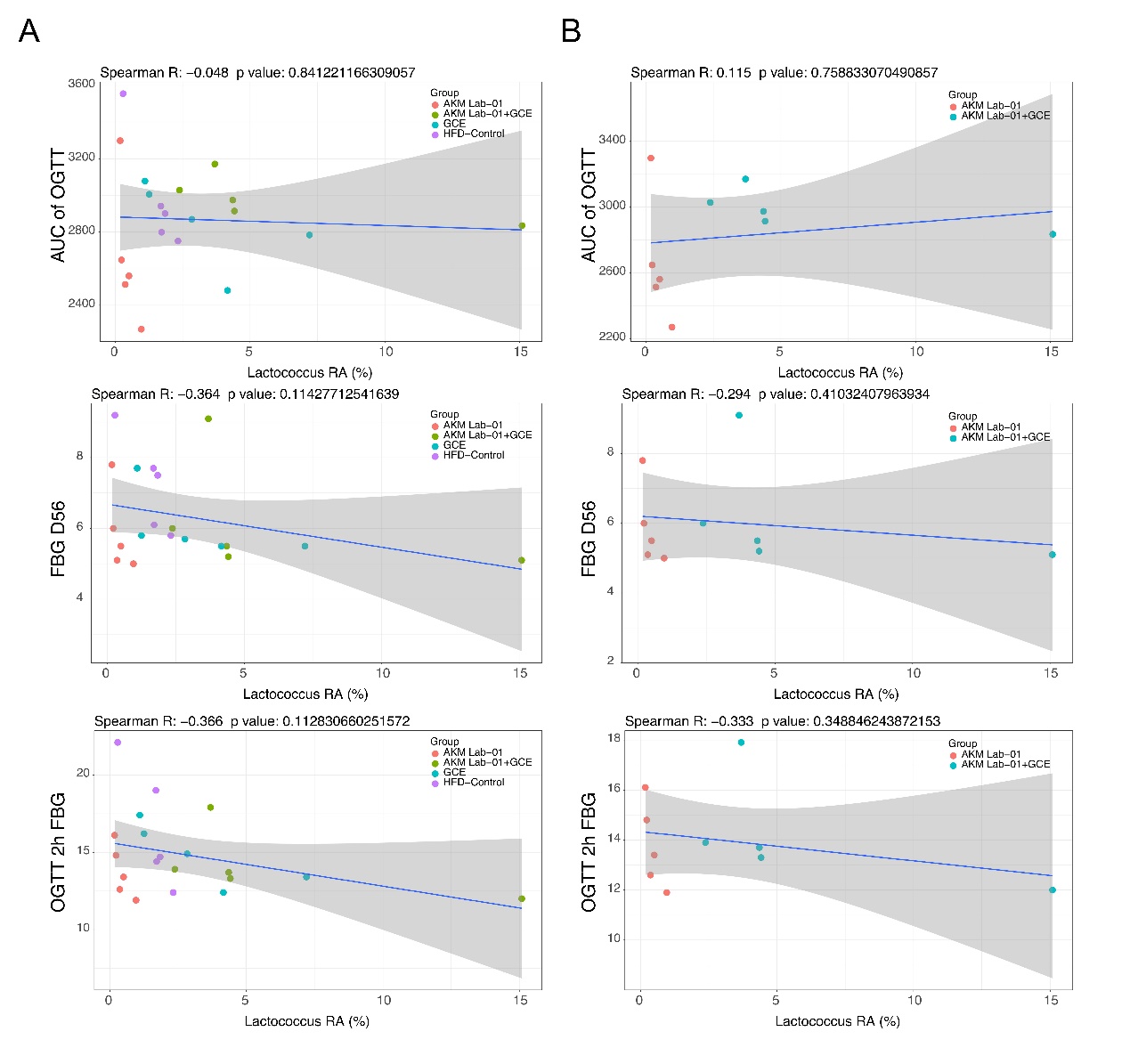


**Figure S3. Correlation analysis between** ***Lactococcus* abundance and glucose parameters. A.** Pooled analysis across all experimental groups. **B.** Analysis between the AKM monotherapy and combination-treatment group.
